# Triacylglycerol Fatty Acid Composition in Diet-Induced Weight Loss in Subjects with Abnormal Glucose Metabolism – the GENOBIN Study

**DOI:** 10.1371/journal.pone.0002630

**Published:** 2008-07-09

**Authors:** Ursula Schwab, Tuulikki Seppänen-Laakso, Laxman Yetukuri, Jyrki Ågren, Marjukka Kolehmainen, David E. Laaksonen, Anna-Liisa Ruskeepää, Helena Gylling, Matti Uusitupa, Matej Orešič, for the GENOBIN Study Group

**Affiliations:** 1 School of Public Health and Clinical Nutrition, Department of Clinical Nutrition and Food and Health Research Centre, University of Kuopio, Kuopio, Finland; 2 Kuopio University Hospital, Kuopio, Finland; 3 VTT Technical Research Centre of Finland, Espoo, Finland; 4 Department of Physiology, University of Kuopio, Kuopio, Finland; 5 Department of Medicine, Kuopio University Hospital, Kuopio, Finland; Uppsala University, Sweden

## Abstract

**Background:**

The effect of weight loss on different plasma lipid subclasses at the molecular level is unknown. The aim of this study was to examine whether a diet-induced weight reduction result in changes in the extended plasma lipid profiles (lipidome) in subjects with features of metabolic syndrome in a 33-week intervention.

**Methodology/Principal Findings:**

Plasma samples of 9 subjects in the weight reduction group and 10 subjects in the control group were analyzed using mass spectrometry based lipidomic and fatty acid analyses. Body weight decreased in the weight reduction group by 7.8±2.9% (*p*<0.01). Most of the serum triacylglycerols and phosphatidylcholines were reduced. The decrease in triacylglycerols affected predominantly the saturated short chain fatty acids. This decrease of saturated short chain fatty acid containing triacylglycerols correlated with the increase of insulin sensitivity. However, levels of several longer chain fatty acids, including arachidonic and docosahexanoic acid, were not affected by weight loss. Levels of other lipids known to be associated with obesity such as sphingolipids and lysophosphatidylcholines were not altered by weight reduction.

**Conclusions/Significance:**

Diet-induced weight loss caused significant changes in global lipid profiles in subjects with abnormal glucose metabolism. The observed changes may affect insulin sensitivity and glucose metabolism in these subjects.

**Trial Registration:**

ClinicalTrials.gov NCT00621205

## Introduction

Dyslipidemia and abnormal fatty acid metabolism - elongation and desaturation - are characteristic in obesity, especially in association with the metabolic syndrome and abnormal glucose metabolism, *i.e*. impaired fasting glucose (IFG), impaired glucose tolerance (IGT) and type 2 diabetes (T2DM) [1]. Weight loss improves the serum lipid profile in part by decreasing the concentration of serum triacylglycerols (TG) and increasing HDL cholesterol and HDL/LDL ratio in an antiatherogenic direction [2]–[4].

The effect of weight loss on specific plasma lipid molecular subclasses is not well understood. Such information may be valuable to elucidate specific lipid species that directly or indirectly affect insulin sensitivity. For example, elevated flux of saturated fatty acids (*e.g.*, palmitic or stearic acid) into peripheral tissues may lead to production of lipotoxic metabolites in peripheral tissues [5]–[7]. Changes in the fatty acid composition of TG, the major carriers of fatty acids to peripheral tissues, may therefore alter the risk for developing lipotoxicity-related complications.

Obesity and insulin resistance are associated with impaired elongation and desaturation of fatty acids, reflected in the serum by, *e.g.*, higher proportions of myristic and palmitic acid and lower proportions of longer-chain n-6 and n-3 fatty acids [8], [9]. Such an adverse fatty acid profile has also predicted worsening insulin resistance, hyperglycemia and T2DM in prospective cohort studies [10]–[12]. Diet-induced weight loss has resulted in desaturation and elongation of serum and tissue fatty acids in addition to an improvement in insulin sensitivity [13].

Sphingomyelin, a marker of abnormal sphingolipid metabolism and a possible risk factor in atherosclerosis [14], has also been elevated in an obese mouse model [15]. Lysophosphatidylcholines (LPC), phospholipase A2-generated hydrolysis products of phosphatidylcholines (PC), regulate a range of pro-inflammatory molecules [16]. They have been elevated in patients with T2DM [17], atherosclerosis [18] and obesity [19]. Levels of LPC also positively correlate with measures of subcutaneous obesity and negatively with insulin sensitivity [19].

Analytical platforms and informatic tools have been recently developed that afford extended and sensitive measurement of the lipidome [20], [21]. Current lipidomics platforms enable quantitative characterization of hundreds of diverse lipid molecular species across multiple lipid classes, including sphingolipids, phospholipids (PL), sterol esters, and acylglycerols [21]–[27]. The exact fatty acid composition for each lipid can usually be determined. It is therefore possible to broadly characterize the effect of weight loss on plasma lipidome, specifically in relation to features of energy storage and signaling associated with the metabolic syndrome and insulin resistance.

We hypothesized that the diet induced weight loss affects the global plasma lipidomic profile, and that the observed profile changes may provide new clues about the physiology of weight change. The lipidomics strategy was applied to examine what changes in lipidomic profile occur after a successful diet-induced weight loss according to current dietary recommendations in middle-aged and older (elderly over 75 years of age) subjects with IFG or IGT and the metabolic syndrome in a 33-week trial [28].

## Methods

The original protocol for this trial (in Finnish) and the CONSORT checklist are available as supporting information, Protocol S1 and Checklist S1.

### Participants

Altogether 75 overweight or obese (BMI 28–40 kg/m^2^) subjects aged 40 to 70 years were recruited to the GENOBIN study [28]. The subjects had IFG (fasting plasma glucose concentration 5.6–7.0 mmol/l) or IGT (2-hour plasma glucose concentration 7.8–11.0 mmol/l) and at least 2 other features of the metabolic syndrome according to the Adult Treatment Panel III criteria [29]: waist circumference >102 cm (males)/>88 cm (females), fasting serum TG concentration ≥1.7 mmol/l, fasting serum HDL-cholesterol <1.0 mmol/l (males)/<1.3 mmol/l (females), blood pressure ≥130/85 mmHg.

### Objectives

The aim of this study was to examine whether a diet-induced weight reduction and dietary changes towards current recommendations result in changes in the extended plasma lipid profiles (lipidome) in subjects with impaired fasting glucose (IFG) or impaired glucose tolerance (IGT) and features of metabolic syndrome in a 33-week intervention.

### Ethics

The intervention was performed in accordance with the principles of the Declaration of Helsinki. The Ethics Committee of the District Hospital Region of Northern Savo and Kuopio University Hospital approved the study plan, and all participants gave their written informed consent.

### Interventions

Subjects were randomly assigned to a weight reduction (WR) group (*n* = 28), resistance training group (*n* = 14), aerobic exercise group (*n* = 15) or a control group (*n* = 18). Subjects were matched for age, gender, BMI and the status of glucose metabolism (IFG/IGT). At screening, the health status and medical history of the subjects were examined by an interview, clinical examination, and laboratory examinations including measurements for liver, kidney and thyroid functions. The duration of the study was 33.1±2.0 weeks (mean±SD). The WR group had a 12-week intensive WR period during which they met a clinical nutritionist for five times. A clinical nutritionist gave individual instructions based on an interview and 4-day food records kept by the subjects three times during the intensive period (at weeks 1, 7–8 and 12), aiming to decrease the energy intake level and meet the American Heart Association criteria [30]. The minimum aim for study weeks 12–33 was to maintain the reduced body weight achieved. The subjects were asked to maintain their habitual level of physical exercise throughout the intervention period. During the weeks 12-33 the subjects kept 4-day food records twice (22 weeks and 32 weeks). The subjects in the control group were advised to continue their normal lifestyle during the study, and to keep their diet and exercise habits unchanged. Food records were kept at weeks 0, 12 and 32.

**Figure 1:**
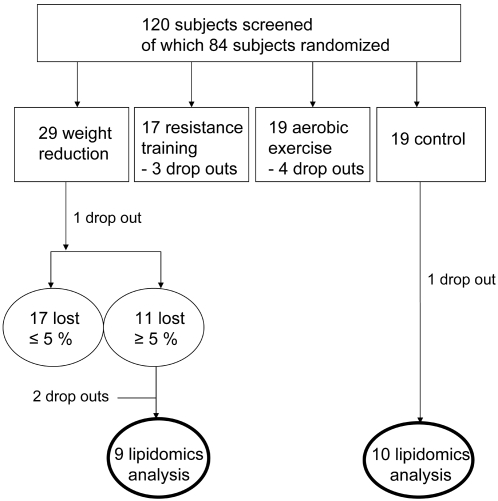
CONSORT chart showing selection of subjects for lipidomic analysis.

Lipidomics analysis was performed in those subjects in the WR and control groups whose adipose tissue samples were chosen for the transcriptomic analysis [28]. Those subjects were chosen based on the weight change during the intervention (Figure 1). The inclusion criteria in the WR group was >5% from the baseline weight and ±2 kg maximum in the control group. Initially, there were 10 subjects chosen in the WR group, but one subject was excluded due to rheumatoid arthritis. The baseline characteristics of these 19 subjects are presented in Table 1.

**Table 1:** Baseline characteristics of the subjects.

	Weight reduction (N = 9)	Control (N = 10)	*p*-value*
Gender (males/females)	4/5	4/6	
Age (y)	58±7	62±8	NS
Weight (kg)	97.8±19.8	86.6±6.6	NS
BMI (kg/m^2^)	34.2±4.3	31.8±2.6	NS
Blood pressure (mmHg)			
Systolic	140±14	135±9	NS
Diastolic	95±7	84±7	0.001
Waist circumference (cm)	111±12	104±7	NS
Body fat (%)	39.7±6.8	38.8±7.4	NS
Serum cholesterol (mmol/l)			
Total	5.4±1.1	4.9±0.7	NS
HDL	1.12±0.17	1.29±0.17	NS
LDL	3.61±0.91	3.10±0.52	NS
VLDL	0.69±0.36	0.51±0.27	NS
Serum triglycerides (mmol/l)	1.81±0.84	1.61±0.57	NS
S_I_; insulin sensitivity index (mU/l)^−1^min^−1^	1.78+0.42	2.66+0.97	0.027

* Mann-Whitney's U-test for independent samples.

### Outcomes

#### Anthropometric measures

Body weight was measured with a standardized electronic scale in light clothing. Height was measured at the beginning of the study in a Frankfurt position. The waist circumference was measured halfway between the lowest rib and the iliac crest. Body composition was measured by a bioelectrical impedance STA/BIA Body Composition Analyzer (Akern Bioresearch Srl, Florence, Italy).

#### Biochemical measures

Blood samples for biochemical analyses were drawn at weeks 0 and 33 after a 12 hour overnight fast from an antecubital vein while a subject was in a sitting position. Biochemical analyses were performed using routine methods in Clinical Laboratory Centre of the Kuopio University Hospital and in the Clinical Unit of the University of Kuopio. For the analysis of serum concentrations of lipoprotein lipids, lipoproteins were separated by ultracentrifucation (Beckman Optima L-90K, Palo Alto, Ca). Enzymatic colorimetric methods with commercial kits (cholesterol: CHOD-PAP, TG: GPO-PAP, Roche Diagnostics GmbH, Mannheim, Germany) were used to determine cholesterol and TG concentrations from whole serum and separated lipoproteins with an automated instrument (Kone Pro Clinical Chemistry Analyzer, Thermo Clinical Labsystems, Konelab, Espoo, Finland).

The frequently sampled intravenous glucose tolerance test (FSIGT) was performed according to the Minimal Model Method as previously described [31]. Insulin sensitivity index (S_I_) was calculated by the MINMOD Millennium software [32].

#### Fatty acid analysis

Lipids from serum samples (40 µl) were extracted by chloroform∶methanol (2∶1, 200 µl) after addition of heptadecatrienoate [TG(17:0/17:0/17:0)] as internal standard. The lower layer was separated, evaporated into dryness under nitrogen flow and dissolved into petroleum ether (bp. 40–60°C). Bound fatty acids were transmethylated with sodium methoxide (NaOMe, 0.5M in methanol) by boiling at 45°C for 5 min. The mixture was acidified by adding NaHSO_4_ (15% solution) and extracted with petroleum ether [33]. The organic phase containing fatty acid methyl esters (FAME) and free fatty acids (FFA) was separated into a glass vial, evaporated under nitrogen, redissolved into hexane (50 µl) and transferred into a glass insert for GC analysis.

Two µl aliquots were used for GC injection (split ratio 1∶20) at 260°C. The Agilent 5890 series II GC was equipped with a 25 m FFAP capillary column (i.d. 0.32 mm) and helium was used as carrier gas (30 ml/min). The oven temperature was increased from 70°C to 240°C at 7°C/min and the fatty acids detected by flame ionization detector (FID). Identification was based on retention times and GC/MS spectra of known standards as well as on literature.

#### Lipidomics

The lipidomics platform based on Ultra Performance Liquid Chromatography (UPLC) coupled to high resolution mass spectrometry (MS) was utilized. The applied platform affords broad screening of multiple lipid classes from total lipid extracts within a single sample run. Because profile changes across a diverse range of lipids such as PL and neutral lipids were of interest, the experiment was performed in positive ion mode.

With UPLC, a run time of 12 minutes can be achieved without significant loss in sensitivity, covering major monoacylglycerols and PL, diacylglycerols and diacylglycero-PL, sphingolipids, TG, and cholesterol esters. The platform has already been applied in multiple clinical and preclinical studies [7], [19], [34], [35]. The analytical method utilized is the same as recently reported [34], so the UPLC/MS based lipidomics methodology is described below only in brief.

An aliquot (10 µl) of an internal standard mixture containing 11 lipid classes, and 0.05M sodium chloride (10 µl) was added to plasma samples (10 µl). The lipids were extracted with chloroform/methanol (2∶1, 100 µl). A standard mixture containing 3 labeled standard lipids was added (10 µl) to the extracts. The sample order for LC/MS analysis was determined by randomization.

Lipid extracts were analysed on a Waters Q-Tof Premier mass spectrometer combined with an Acquity Ultra Performance LC™ (UPLC). The column, which was kept at 50°C, was an Acquity UPLC™ BEH C18 10×50 mm with 1.7 µm particles. The binary solvent system included A. water (1% 1M NH_4_Ac, 0.1% HCOOH) and B. LC/MS grade (Rathburn) acetonitrile/isopropanol (5∶2, 1% 1M NH_4_Ac, 0.1% HCOOH). The gradient started from 65% A/35% B, reached 100% B in 6 min and remained there for the next 7 min. The total run time including a 5 min re-equilibration step was 18 min. The flow rate was 0.200 ml/min and the injected amount 0.75 µl. The temperature of the sample organizer was set at 10°C.

The lipid profiling was carried out on Waters Q-Tof Premier mass spectrometer using ESI+ mode. The data were collected at mass range of m/z 300–1200 with a scan duration of 0.2 sec. The source temperature was set at 120°C and nitrogen was used as desolvation gas (800L/h) at 250°C. The voltages of the sampling cone and capillary were 39 V and 3.2 kV, respectively. Reserpine (50 µg/L) was used as the lock spray reference compound (5 µl/min; 10 sec scan frequency).

Data were processed using MZmine software version 0.60 [36]. Lipids were identified using internal spectral library or with tandem mass spectrometry as described previously [21]. The normalization of lipidomics data was performed as follows: All monoacyl lipids except cholesterol esters, such as monoacylglycerols and monoacylglycero-PL were normalized with GPCho(17:0/0:0), all diacyl lipids except ethanolamine PL were normalized with GPCho(17:0/17:0), ceramides with Cer(d18:1/17:0), the diacyl ethanolamine phospholipids were normalized with GPEtn(17:0/17:0), and the TG and cholesterol esters with TG(17:0/17:0/17:0). Other (unidentified) molecular species were calibrated with GPCho(17:0/0:0) for retention time <300 seconds, GPCho(17:0/17:0) for retention time between 300s and 410s, and TG(17:0/17:0/17:0) for higher retention times.

### Statistical methods

The statistical analyses of biochemical data were performed using the SPSS statistical software (v. 14.0.1, SPSS Inc., Chicago, IL). The normality of the distributions of the variables was verified with the Kolmogorov-Smirnov test with Lilliefors' correction. Mann Whitney's *U*-test was used to compare the groups at baseline. The General Linear Model (GLM) for repeated measurements was used for analyzing the interaction of time and group. The paired samples *t*-test was used to compare the changes within the groups and an independent samples *t*-test was used for comparisons between the groups. Variables with abnormal distribution were analyzed by Wilcoxon matched pairs signed ranks test (within the groups) and Mann Whitney's *U*-test (between the groups). The data are expressed as mean±SD, and a *p*-value <0.05 was considered as statistically significant.

The paired samples *t*-test was used to compare the lipid molecular species level changes within the groups, and two-sided unpaired *t*-test was applied to compare the control and intervention groups at a specific time point. In order to correct for multiple comparisons, False Discovery Rate (FDR) *q*-value was calculated [37], with significance threshold set at *q*<0.05. Spearman correlation was applied to calculate *r* correlation coefficient, with *p*-value testing the hypothesis of no correlation against the alternative that there is a nonzero correlation using the large-sample approximation. The box plots used to visualize lipid levels within the groups should be interpreted as follows: The box itself contains the middle 50% of the data. The upper edge (hinge) of the box indicates the 75th percentile of the data set, and the lower hinge indicates the 25th percentile. The line in the box indicates the median value of the data. The ends of the vertical lines or “whiskers” indicate the minimum and maximum data values, unless outliers are present in which case the whiskers extend to a maximum of 1.5 times the inter-quartile range. The points outside the ends of the whiskers are outliers or suspected outliers.

## Results

In the WR group the change in body weight was −7.8±2.9% (−7.7±3.3 kg) (*p*<0.01, week 33 *vs*. baseline), whereas in the control group the change was 0.3±1.2% (0.3±1.1 kg) (*p* <0.001, WR *vs*. control group). The change in S_I_ was 32.3±41.9% in the WR group and 6.3±52.1% in the control group (*p* = 0.074, WR vs. control group). Fasting plasma glucose concentrations decreased 6.9±9.7% in the WR group and 1.6±11.0% in the control group (*p* = 0.140 in GLM).

The food record data suggested that the decrease in the energy intake was mostly due to the decrease in the intake of total and saturated fat. However, there were no statistically significant changes in the intake of total fat (34.7±6.5 vs. 31.9±5.4, 0 *vs.* 32 weeks, respectively), or saturated (13.1±3.0 *vs.* 11.5±2.0), monounsaturated (11.5±2.5 vs. 11.2±2.6) or polyunsaturated fatty acids (6.2±1.6 *vs*. 5.7±1.5) in the WR group as % of energy (E%). The respective values for the control group were: total fat 32.4±9.8 *vs.* 32.7±5.4, saturated fat 12.7±4.1 *vs.* 12.5±2.0, monounsaturated fat 10.5±3.9 *vs.* 11.2±2.4, and polyunsaturated fat 5.1±1.8 *vs.* 5.1±1.3 E%, 0 *vs.* 32 weeks, respectively, *p* = NS. The changes in the intake of total fat or fatty acid groups did not differ between the groups. The intake of energy was 7096±2160 kJ *vs.* 6092±702 kJ in the WR group, 0 *vs.* 32 weeks, respectively, and 7923±1537 kJ *vs.* 7441±1421 kJ in the control group, 0 *vs.* 32 weeks, respectively. At 32 weeks the energy intake differed between the groups (*p* = 0.02).

Concentrations of serum total and lipoprotein lipids did not change significantly during the intervention and did not differ between the groups, partly due to the small sample size. The mean change in serum total cholesterol concentrations during the study were 0.38±10.91% in the WR group and 3.16±7.80% in the control group (*p* = NS). The respective values for serum concentration of total TG were −13.33±33.32% in the WR group and −5.02±20.36% in the control group (*p* = NS). Regarding the changes in serum HDL cholesterol concentration the respective values were 8.14±13.75% in the WR group and −0.80±10.31% in the control group (*p* = NS). There were no significant changes in serum total-to-HDL cholesterol ratio or TG-to-HDL cholesterol ratio during the study.

**Figure 2:**
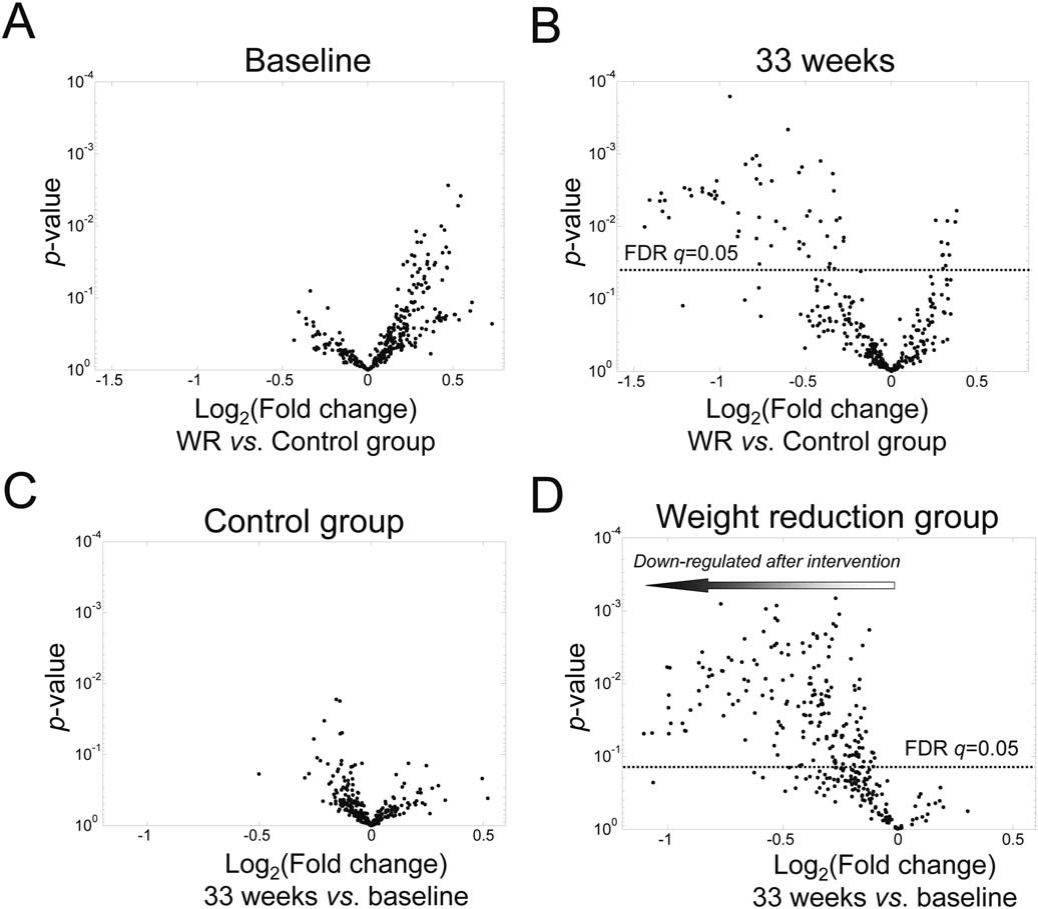
Fold changes and *t*-test *p*-values between mean concentrations in the weight reduction and control groups at (A) baseline and (B) after the intervention for the 309 peaks, among them180 identified lipid molecular species. Also shown are fold changes between lipid levels after (33 weeks) *vs.* before (0 weeks) the intervention and corresponding paired *t*-test *p*-values for the (C) control group and (D) weight reduction group. The ‘volcano plot’ arranges lipids along dimensions of biological and statistical significance. The first (horizontal) dimension is the fold change between the two groups (on a log scale, so that up and down regulation appear symmetric), and the second (vertical) axis represents the *p*-value for a *t*-test of differences within the group after and before the intervention. The line above which False Discovery Rate *q*-value <0.05 is marked. No lipid passed the *q*<0.05 treshold in panels A and C.

To assess the lipid profiles at the molecular species level, the lipidomic analysis was performed using the UPLC/MS platform. Following data processing and quality control, a total of 309 lipids were detected, of which 180 lipid molecular species were identified. When comparing the average lipid levels between the WR and control groups at baseline, none of the lipids differed significantly after accounting for multiple comparisons (Figure 2A). Most of the lipids found elevated in the WR group at baseline (at *p*<0.05 using the two-sided unpaired *t*-test, but none at FDR *q*<0.05) were sphingomyelins (Table 2). A large number of lipids were found significantly diminished in the WR group when comparing the lipid levels between the WR and control groups after the intervention (Figure 2B).

**Table 2:** Top 20 ranking lipid peaks, based on two-sided unpaired *t*-test statistic comparing baseline differences between the WR and control groups.

Retention time (s)	mass-to-charge ratio (m/z)	Identity	Fold (WR vs. Ctr)	*p*-value	FDR *q*-value*
378	732.6122		1.39	0.003	0.28
419	817.7092	SM(d18:0/24:0)	1.46	0.004	0.28
407	809.6526	SM(d18:1/24:3)	1.44	0.005	0.28
378	731.6084	SM(d18:1/18:0)	1.35	0.010	0.28
407	788.6706		1.37	0.011	0.28
371	768.5899	GPCho(O-36:4)	1.22	0.012	0.28
463	666.6169	ChoE(18:2)	1.26	0.013	0.28
360	704.5767		1.24	0.017	0.28
371	794.6037	GPCho(O-38:5)	1.22	0.017	0.28
412	801.6835	SM(d18:1/23:0)	1.38	0.020	0.28
357	744.5556	GPEtn(36:2)	1.39	0.023	0.28
399	774.6529		1.36	0.024	0.28
384	796.6191	GPCho(O-38:4)	1.28	0.025	0.28
360	703.5732	SM(d18:1/16:0)	1.20	0.025	0.28
371	795.6085		1.21	0.026	0.28
407	787.6665	SM(d18:1/22:0)	1.27	0.031	0.28
380	787.6025		1.17	0.031	0.28
419	815.6985	SM(d18:1/24:0)	1.32	0.032	0.28
399	800.6717		1.27	0.034	0.28
378	740.6084		1.16	0.034	0.28

* Computed using algorithm by Storey et al. [37].

When comparing within-person changes between the baseline and after the intervention in each group separately, only modest fluctuations of the lipid levels were found in the control group, as revealed by the volcano plot (Figure 2C), and none of the changes were found significant after adjusting for multiple comparisons using FDR. In contrast, a large number of lipid molecular species decreased in the WR group (Figure 2D), with 175 out of 309 lipids having FDR *q*-value <0.05.

**Figure 3:**
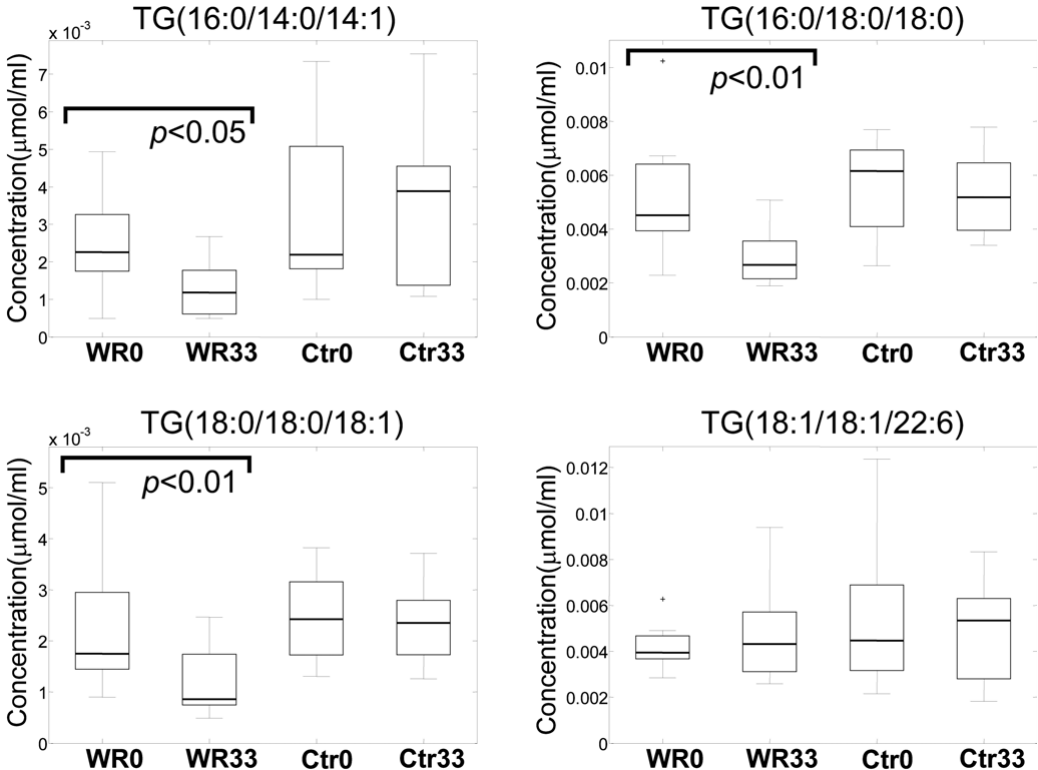
Box plots for four selected triacylglycerol (TG) lipids in the weight reduction group before (WR0) and after (WR33) intervention, as well as for the control group before (Ctr0) and after (Ctr33) intervention. The fatty acid composition of the individual acyls of the respective TG particles are shown in parentheses.

The major general trend observed was down-regulation of most of the TG, PC, and phosphatidylethanolamines in the WR group. A few representative TG species levels in different groups are shown in Figure 3. Interestingly, the level of the docosahexaenoic acid-containing long-chain TG species [TG(18:1/18:1/22:6)] did not change in the WR group.

**Figure 4:**
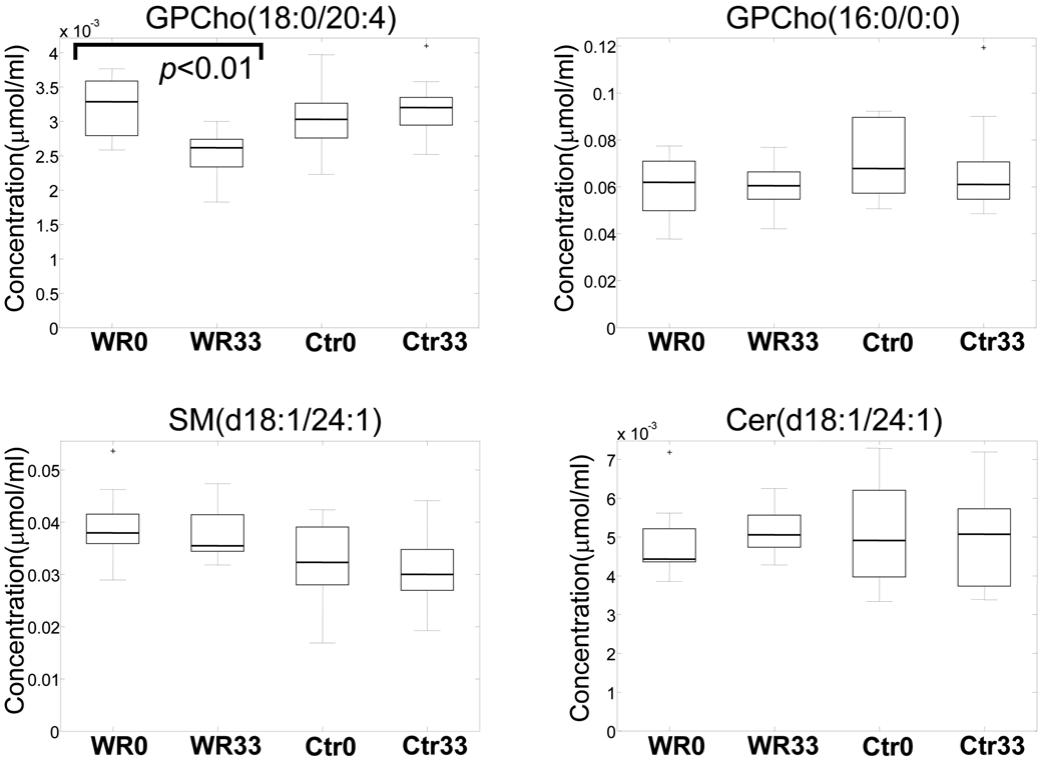
Box plots for selected phospho- and sphingolipids in the weight reduction (WR) and control (Ctr) groups at the beginning (WR0, Ctr0) and at the end (WR33, Ctr33) of the study. GPCho, phosphatidylcholine; SM, sphingomyelin; Cer, ceramide.

However, the trend for reduced lipid levels did not affect all lipid classes (Figure 3). While PCs were reduced (Figure 4 shows an example of one representative PC species, GPCho(18:0/20:4)), the levels of LPC (*e.g.*, GPCho(16:0/0:0), the most abundant LPC in serum), PLA_2_-generated hydrolysis products of PC, did not change. Similarly, the levels of sphingomyelins and ceramides did not change significantly after 33 weeks of diet intervention despite improvement in insulin sensitivity (examples of two abundant sphingolipids, SM(d18:1/24:1) and Cer(d18:1/24:1), are shown in Figure 4).

**Figure 5:**
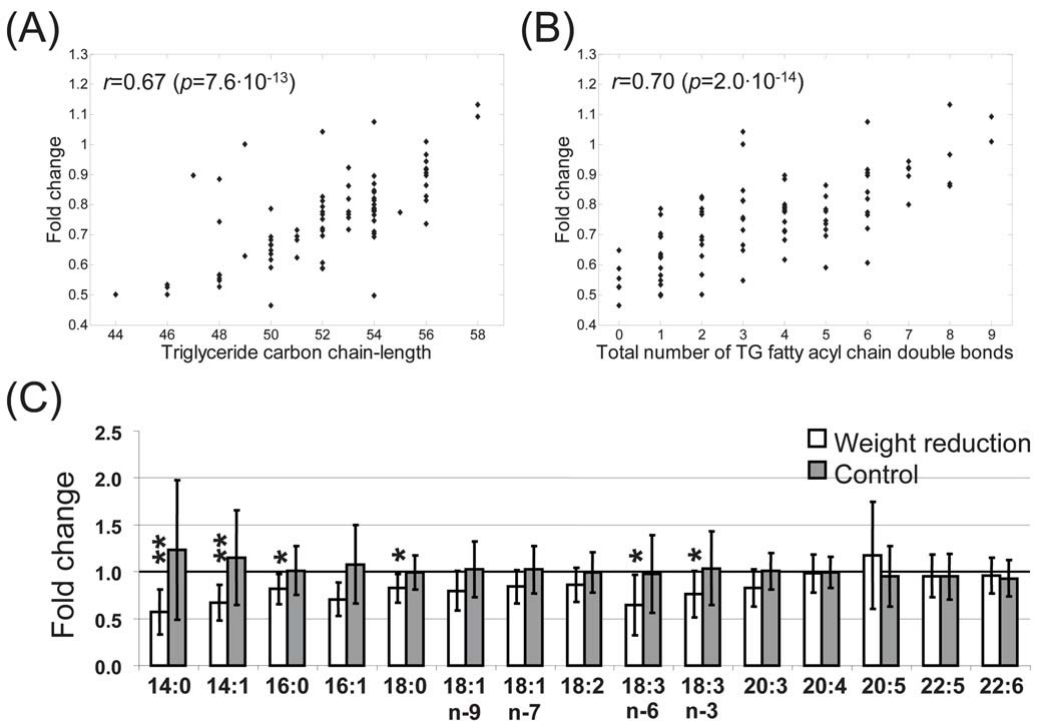
Spearman rank correlation between the fold change of triacylglycerol molecular species after and before the intervention in weight reduction group, and the corresponding (A) total TG fatty acid carbon chain length and (B) degree of TG fatty acid unsaturation. (C) Changes in esterified fatty acid composition in weight reduction and control groups, as obtained by gas chromatography based fatty acid measurement. *p*-values were calculated using paired samples *t*-test (*p<0.05, **p<0.01).

**Figure 6:**
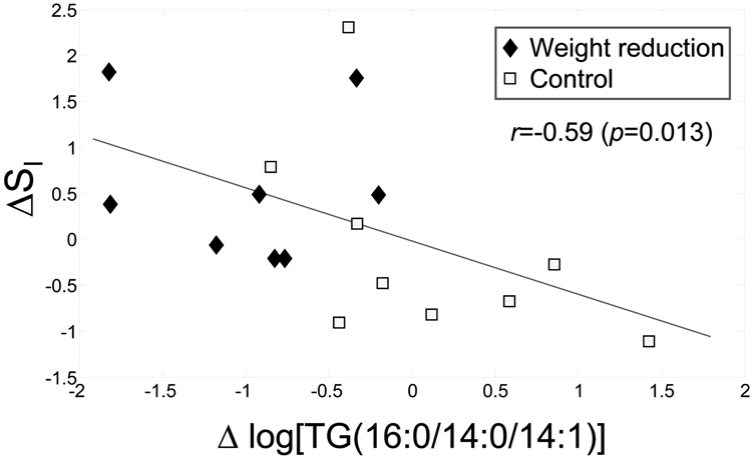
Change in insulin sensitivity index S_I_ (33 weeks *vs.* 0 weeks) *vs.* log-change of triacylglycerol TG(16:0/14:0/14:1) concentration. The Spearman rank correlation was used to calculate correlation coefficient *r*. The regression line is based on linear regression.

Results shown in Figure 3 suggest that the degree of level change in WR group varies for different TG species. Both the TG carbon chain length and the degree of unsaturation, *i.e.*, total number of fatty acyl double bonds in a specific TG species, were found to be correlated significantly with the fold change of TG molecular species in the WR group (33 weeks *vs*. baseline) (Figure 5A and B). The degree of decrease of saturated and short chain TG species was generally more pronounced than for longer chain unsaturated species. These findings were further validated by gas chromatography analysis of fatty acids (Figure 5C). The shorter chain and saturated fatty acids were found significantly downregulated, *e.g.*, myristate, myristoleate, palmitoleate, and stearate, while the levels of longer chain unsaturated essential fatty acids did not alter following the diet intervention. The degree of decrease of TGs containing saturated fatty acids (*e.g.*, TGs with the acyl chains 16:0/14:0/14:1, 16:0/16:0/16:0, 18:1/14:0/16:0, 16:0/18:0/16:0, 16:0/16:0/18:1, 18:1/16:0/16:1, 16:0/18:1/18:0) was significantly correlated with improvement in insulin sensitivity. Figure 6 shows the correlation for TG(16:0/14:0/14:1).

## Discussion

This 33-week trial showed that diet-induced weight loss results in marked changes in the lipidomic profile in middle-aged and older men and women with IFG or IGT, and insulin resistance, and the metabolic syndrome. These changes occurred even in the absence of significant changes in classical lipid measures. TG and PC levels were reduced, with no significant change in sphingolipid and LPC levels. The degree of decrease of saturated and short chain TG species was generally more pronounced than for longer chain unsaturated species, and the degree of change in saturated and short chain fatty acid-containing TG was proportional to the improvement in insulin sensitivity.

The differences in degree of change of TG species supports the view that diet-induced weight loss primarily affects the metabolism of saturated fatty acids, while the metabolism of other physiologically important fatty acids such as arachidonic acid and docosahexaenoic acid is not altered. The proportions of saturated and polyunsaturated fatty acids in TG, cholesterol ester and phospholipid fractions of the serum are considered to be markers of dietary fatty acid intake [38]. Because the circulation also delivers fatty acids from the GI tract, liver and adipose tissue to peripheral tissues, serum fatty acid composition also reflects that of other tissues [38]. However, obesity and insulin resistance is also associated with increased saturation of serum and tissue fatty acids [10], [11]. Based on indirect measures, obesity seems to impair desaturase and elongase enzyme activity [9]. Consistent with earlier studies [13], our findings show that diet-induced weight loss decrease the saturation of fatty acids in TG. Even though the change in the proportion of dietary saturated and polyunsaturated fat did not differ significantly between the WR and control groups, the decrease in the saturation of TG may be a consequence of changes in dietary fatty acid composition, weight loss, or more likely, both.

Moreover, we found that the decrease in the saturation of TG during the trial was associated with the improvement in insulin sensitivity. Our findings are supported by epidemiological studies of TG and serum fatty acid composition and its association with the development of worsening glycemia, insulin resistance, the metabolic syndrome and T2DM [10]–[12], [39], [40]. Serum fatty acid composition predicts development of impaired fasting glycaemia and diabetes in middle-aged men [41]. In dietary trials, replacement of saturated fatty acids with polyunsaturated fatty acids has improved insulin sensitivity and decreased abdominal obesity [41], whereas substituting saturated fatty acids for unsaturated fatty acids results in insulin resistance [42]. Because weight loss itself may alter the fatty acid composition of serum and tissue, decreased saturation and elongation of fatty acids may be one of the mechanisms by which weight loss improves insulin sensitivity. Decreased circulation of saturated fatty acids can also be considered as beneficial because increased flux of saturated fatty acids into peripheral tissues can lead to accumulation of toxic lipid molecular species in these tissues [7], [43], [44].

Our methodology also allowed determination of the fatty acid composition of the acyl chains of whole TG particles. Other studies assessing the role of TG fatty acid composition in obesity, insulin resistance and weight loss have measured the concentrations of the individual fatty acid chains after separation of the TG particles. It is not yet clear whether the measurement of the fatty acid composition of whole TG particles will provide additional physiologically relevant information over older methods.

Most of the common PL such as PC and phosphatidylethanolamines also decreased. This can be explained by the fact that these PL are the major components of TG-carrying lipoproteins such as VLDL, and their concentrations mirror TG levels also postprandially [45], [46].

LPC, lipids found in proinflammatory [47], [48] and proatherogenic conditions [49], did not decrease in the WR group. We have found previously that obesity in weight-discordant monozygous twins was primarily associated with elevated LPCs [19]. The lack of change in LPC levels may in part be explained by the fact that the weight reduction was not very dramatic or the complexity of LPC metabolism. LPC is a major component of oxidized LDL [50]. However, in healthy individuals LPC is most abundant in the HDL fraction [51], and there is a high degree of LPC exchange between HDL and oxLDL [52]. It is therefore possible that LPC levels of oxLDL may in part be balanced by elevated HDL [51], [52]. Analyses of LPC levels in lipoprotein subfractions will therefore be needed in future studies to provide better understanding of LPC regulation during diet-induced weight loss.

Sphingomyelins and ceramides did not change following weight loss. In a recent study, plasma sphingomyelins and ceramides were elevated in the plasma of genetically obese ob/ob mice [15]. However, in the monozygous twin study we found that in particular very-long-chain fatty acid-containing (*e.g.*, nervonic acid, 24∶1) sphingomyelins are remarkably similar within obesity-discordant twin co-pairs [19]. Therefore, it is plausible that the genetic component involved in regulation of systemic sphingolipid metabolism is more important than the effects of obesity and weight loss.

As a potential weakness of the study, the clinical characteristics of the two groups were not entirely similar at the baseline. The insulin sensitivity index S_I_ was higher in the control group than in the WR group. As sphigomyelins are known to correlate with fasting serum insulin [19], this may explain the observed trend towards the elevated sphingomyelins in the WR group at baseline (Table 2). Sphingomyelins were also modestly elevated in the WR group after the intervention, but none of the differences reached *p*<0.05. Although the weight reduction associated changes of triacylglycerols are well supported by the data, we cannot exclude the possibility that the observed baseline differences in specific lipid classes, as well as small sample size, may have masked other potential changes across different lipid classes. This clearly needs to be investigated in further studies.

In conclusion, we demonstrated that diet-induced weight loss led to a remarkable alteration of global lipid profiles in subjects with impaired fasting glucose or impaired glucose tolerance. These changes were dominated by a reduction in saturated and short-chain fatty acid-containing triacylglycerols, which was coupled with an improvement in insulin sensitivity. Our data also indicate that lipidomics technology can complement classical clinical lipid measures and can provide valuable clues about physiologically relevant changes in lipid metabolism, even when classical lipid measures are not significantly altered.

## Supporting Information

Protocol S1 Trial Protocol. Original GENOBIN study plan (in Finnish). The study plan was accepted by the Ethics Committee of the Hospital District of Northern Savo (acceptance# in public records: 152/2002).
(0.18 MB PDF)

Checklist S1 CONSORT Checklist
(0.19 MB PDF)
