# Supplementary material for: Triacylglycerol Fatty Acid Composition in Diet-Induced Weight Loss in Subjects with Abnormal Glucose Metabolism – the GENOBIN Study
Source: PLoS One. 2008 Jul 9;3(7):e2630. doi: 10.1371/journal.pone.0002630 (PMC2440352; doi:10.1371/journal.pone.0002630)
Supplement: Protocol S1 — Trial Protocol. Original GENOBIN study plan (in Finnish). The study plan was accepted by the Ethics Committee of the Hospital District of Northern Savo (acceptance# in public records: 152/2002). (0.18 MB PDF) [file pone.0002630.s001.pdf]

20.11.02

GEENIEN ILMENTYMINEN RASVAKUDOKSESSA LIHAVUUDESSA JA INSULIINIRESISTENSSISSÄ

tutkimusryhmän johtaja, professori Matti Uusitupa
tutkimusjohtaja, FT Leena Pulkkinen
tutkija, THM Marjukka Kolehmainen
tutkijakoulutettava, LL Katariina Sivenius
tutkijakoulutettava, FM Titta Salopuro
tutkijakoulutettava, FM Niina Siitonen
tutkijakoulutettava, THM Virpi Lindi

TAUSTA

Lihavuus yleistyy koko ajan maailmanlaajuisesti (WHO, 2000), Suomi mukaan lukien. Suomessa runsas puolet väestöstä on ainakin lievästi liikapainoisia, ja merkittävästi lihavia keski-ikäisessä väestössä on peräti viidennes (Puska *et al.*, 1996; Lahti-Koski *et al.*, 2000). Hälyttävää on, että aikuisten lisäksi lihavuus on myös lasten ja nuorten ongelma (Ebbeling *et al.*, 2002). Lihavuus altistaa useille kroonisille sairauksille, kuten tyypin 2 diabetekselle ja sepelvaltimotaudille (Kopelman, 2000), ja tätä kautta lihavuus vaikuttaa myös kansantaloudellisesti lisäten huomattavasti terveydenhuollon kustannuksia. Lähitulevaisuudessa ei ole nähtävissä, että väestön painon kehitys kääntyisi edulliseen suuntaan, ellei lihavuuden merkitykseen terveyden vaarantajana, sen aiheuttamiin sairauksiin ja lihavuuden ehkäisyyn kiinnitetä huomiota sekä väestö- että yksilö-tasolla.

Lihavuuden taustalla vaikuttavat monet tekijät, ja tavallinen lihavuus on useiden ympäristötekijöiden ja perintötekijöiden sekä niiden yhdysvaikutusten seurausta (Kopelman, 2000). Tiedetään, että ravinnon komponentit voivat vaikuttaa perintötekijöiden toimintaan solutasolla, ja toisaalta perintötekijöiden ilmentymisen seurauksena syntyneet proteiinit vaikuttavat ravintoaineiden imeytymiseen, niiden aineenvaihduntaan elimistössä ja energiantuottoon. Toistaiseksi geenien ilmentymisestä ja geenien ja ruokavalion yhdysvaikutuksista ei ole juuri tutkimustietoa johtuen osittain puutteellisista tutkimusmenetelmistä. DNA-mikrosirutekniikan keksiminen ja molekyylibiologisten menetelmien kehittyminen ovat kuitenkin mahdollistaneet geenien ilmentymisen tutkimisen lähes koko genomin laajuudessa.

Rasvakudos on osoittautunut viimeaikaisten tutkimusten perusteella aktiiviseksi kudokseksi, joka osallistuu energia-, glukoosi- ja insuliiniaineenvaihdunnan säätelyyn tuottamalla eräitä merkittäviä geenien ilmentymiseen vaikuttavia tekijöitä, kuten peroksisomi proliferaattoreilla aktivoituva reseptori gammaa (PPAR-γ) sekä sterolien säätelykohtaan sitoutuvaa proteiinia (sterol regulatory element binding protein, SREBP) (Rocchi & Auwerx, 1999). Lisäksi rasvakudoksesta vapautuu verenkiertoon mm. leptiiniä, resistiiniä, adiponektiiniä ja sytokiinejä (mm. tuumorinekroositekijä alfaa) (Kim & Moustaid-Moussa, 2000), joiden epäillään olevan yhteydessä sekä lihavuuden että insuliiniresistenssin syntyyn. Tämän vuoksi rasvakudoksen tutkiminen on tärkeä osa ei ainoastaan lihavuuden vaan myös tyypin 2 diabeteksen taustalla vaikuttavien tekijöiden selvitystyötä.

Kuopion yliopiston kliinisen ravitsemustieteen laitoksella on tutkittu rasvakudosta ja lihavuutta professori Uusitupan johdolla useasta eri näkökulmasta. Tutkimuskohteena ovat olleet mm.

rasvakudoksen aineenvaihdunta (Kolehmainen *et al.*, 2000; Kolehmainen *et al.*, 2002), lihavuuden ja energia- aineenvaihdunnan genetiikka (Sipiläinen *et al.*, 1997b; Sipiläinen *et al.*, 1997a; Valve *et al.*, 1998; Valve *et al.*, 1999; Lindi *et al.*, 2001) sekä lihavuuden vaikutus eri sairauksiin, kuten tyypin 2 diabetekseen (Sivenius *et al.*, 2000; Sivenius *et al.*, 2001). Kliinisen ravitsemustieteen laitos on myös mukana suuressa suomalaisessa tyypin 2 diabeteksen ehkäisytutkimuksessa (The Finnish Diabetes Prevention Study, DPS), jossa tavoitteena oli selvittää, voidaanko tyypin 2 diabeteksen puhkeaminen estää elämäntapamuutoksilla ylipainoisilla henkilöillä, joilla on heikentynyt sokerinsieto. Tutkimuksessa todettiin, että intensiivisellä ravitsemus- ja elämäntapoihin vaikuttavalla hoidolla oli mahdollista ehkäistä tyypin 2 diabeteksen puhkeamista tai ainakin siirtää sitä (Tuomilehto *et al.*, 2001). DPS-aineisto antaa erinomaisen mahdollisuuden selvittää perintötekijöiden ja perintö- ja elämäntapatekijöiden välisten yhdysvaikutusten merkitystä tyypin 2 diabeteksen ja lihavuuden ehkäisyssä ja hoidossa, ja olemmekin jo aloittaneet tämän tutkimuskokonaisuuden DPS-aineistossa (Lindi *et al.*, 2002). Lisäksi olemme tutkineet geenien ilmentymistä rasvakudoksessa sairaalloisesti lihavilla henkilöillä ennen ja jälkeen laihdutuksen (Kolehmainen *et al.*, 2001; Kolehmainen *et al.*, 2002). Nämä tutkimukset ovat antaneet uutta tietoa lihavuuden ja laihtumisen vaikutuksesta glukoosi- ja insuliiniaineenvaihduntaan osallistuvien geenien säätelyyn.

Aiemmat havaintomme ja alustavat tulokset DPS-tutkimuksen Kuopion osa-aineistosta osoittavat, että tyypin 2 diabetesriskin väheneminen on yhteydessä koehenkilöiden insuliiniherkkyyden lisääntymiseen. Jo pienikin painon lasku DPS-tutkimuksessa liittyi insuliiniherkkyyden parantumiseen. Toistaiseksi ei kuitenkaan tiedetä, mitkä geenit vaikuttavat insuliiniherkkyyden muutoksiin painon muuttuessa. Näiden geenien tunnistaminen ja niiden ilmentymisessä tapahtuvien muutosten tutkiminen antaa uutta tietoa lihavuuden ja tyypin 2 diabeteksen syntymekanismeista. Lisäksi on tärkeää selvittää näissä perintötekijöissä esiintyvien sekvenssimuutosten vaikutus ao. proteiinin rakenteeseen ja toimintaan. Havaitut sekvenssimuutokset saattaisivat osittain selittää mm. sen, miksi jotkut henkilöt ovat alttiimpia lihomaan kuin toiset. Näin ollen lihavuuden ja tyypin 2 diabeteksen riskiryhmään kuuluvat henkilöt voitaisiin tunnistaa aiempaa tarkemmin ja heille voitaisiin suunnitella entistä yksilöllisemmät ruokavalio- ja elämäntapaohjeet. Tämä on sekä kansanterveydellisesti että kansantaloudellisesti tärkeää, koska on ennustettu, että lihavuuden yleisyys kasvaa jatkuvasti ja suomalaisten tyypin 2 diabeetikoiden määrä saattaa jopa kaksinkertaistua seuraavan kymmenen vuoden aikana, jollei tehokasta diabeteksen ehkäisy- tai hoito-ohjelmaa saada kehitettyä.

TUTKIMUKSEN TAVOITTEET

Nyt kyseessä oleva tutkimus on jatkoa sekä DPS-tutkimuksen että tekemiemme geenien ilmentymistä selvittävien tutkimusten tuomille havainnoille. Tämän tutkimuksen tavoitteena on selvittää lihavuuden ja insuliiniresistenssin kannalta keskeisten geenien ja geenialueiden ilmentymistä rasvakudoksessa ennen ja jälkeen laihdutusintervention lihavilla ja heikentyneen sokerinsiedon omaavilla henkilöillä (IGT-potilaat). Valitsemalla tutkimukseen lihavia IGT-potilaita, ja tutkimalla perintötekijöiden ilmentymistä sekä ennen että jälkeen laihdutuksen saadaan uutta tietoa niistä geeneistä, jotka säätelevät lihavuuden syntyä, energia-aineenvaihduntaa ja insuliiniresistenssiä. Vertailuryhmänä tutkitaan normaalin sokerinsiedon omaavia ylipainoisia henkilöitä. Lisäksi samojen geenien tai geenialueiden ilmentymistä tutkitaan normaalipainoisilla, normaalin sokerinsiedon omaavilla henkilöillä

AINEISTON KERUU

Tutkittavien seulonta

Tutkittavat rekrytoidaan mm. lehti-ilmoituksin, työterveydenhuollon sekä KYS:n kliinisen ravitsemuksen yksikön kautta. Tutkimukseen pyritään saamaan 50 ylipainoista IGT-potilasta, 50 normaalin sokerinsiedon omaavaa ylipainoista henkilöä sekä 20 tervettä normaalipainoista vertailuhenkilöä.

Sisäänottokriteerit:

IGT:	BMI 28 – 40 kg/m^2<br>muuttumaton paino viimeisen 3 kk:n aikana<br>paasto glukoosi 6.0 - 6.9 mmol/l (plasma)<br>2-tunnin glukoosi 7.8 – 11.0 mmol/l (plasma)<br>ikä 25 – 60 vuotta
ylipainoiset:	BMI 28 – 40 kg/m^2<br>muuttumaton paino viimeisen 3 kk:n aikana<br>paasto glukoosi $<$ 6.0 mmol/l (plasma)<br>2-tunnin glukoosi $<$ 7.8 mmol/l (plasma)<br>ei muita perussairauksia<br>ikä 25 – 60 vuotta
normaalipainoiset:	BMI 19 – 24.9 kg/m^2<br>muuttumaton paino viimeisen 3 kk:n aikana<br>paasto glukoosi $<$ 6.0 mmol/l (plasma)<br>2-tunnin glukoosi $<$ 7.8 mmol/l (plasma)<br>ei kroonisia sairauksia<br>ikä 25 – 60 vuotta

Seulontaan kutsuttavat henkilöt saavat sekä suullista että kirjallista tietoa tutkimuksesta (liitteet 1 ja 2). Seulontaan ja varsinaiseen tutkimukseen osallistuvilta pyydetään kirjallinen suostumus tutkimukseen osallistumisesta. He voivat kuitenkin vapaasti keskeyttää tai peruuttaa osallistumisensa tutkimukseen milloin haluavat.

Seulontaan osallistuvat kutsutaan yön yli kestäneen paaston jälkeen tutkimukseen Elintarvikkeiden terveysvaikutusten tutkimuskeskukseen (ETTK) Kuopion yliopiston kliinisen ravitsemustieteen laitokselle. Seulontavaiheessa tutkittavien terveydentila selvitetään haastattelulla, verinäytteillä (pieni verenkuva, kokonaiskolesteroli, LDL-kolesteroli, HDL-kolesteroli, triglyseridit, glukoosi, insuliini, TSH ja γ-gt), virtsanäytteellä ja lääkärin tutkimuksella. Lisäksi tehdään oraalinen sokerirasitustesti (OGTT), jossa nautitaan 75 g glukoosia sekoitettuna 3 dl:aan vettä ja otetaan glukoosi- ja insuliininäytteet 0, 60 ja 120 min kuluttua glukoosiliuoksen nauttimisesta. Ne, jotka hyväksytään seulonnan ja sisäänottokriteerien perusteella, kutsutaan varsinaiseen tutkimukseen.

Laihdutusinterventio

Tutkittavat noudattavat aluksi 6 viikkoa erittäin niukasti energiaa sisältävää ruokavaliota (ENE) täysipainoisen valmisteen avulla. ENE-jakson loputtua tutkittavat siirtyvät noudattamaan yksilöllisesti suunniteltua 1200 - 1600 kcal ruokavaliota 3 kuukauden ajan, jonka jälkeen he siirtyvät ns. painon ylläpitovaiheeseen 3 kuukauden ajaksi. Intervention jälkeen tutkittavia seurataan n. 1 vuoden ajan n. 3 kuukauden välein. Intervention aikana tutkittavat käyvät säännöllisesti ETTK:ssa/ kliinisen ravitsemustieteen laitoksella tutkimuskäynneillä, joiden aikana he saavat opastusta ruokavalion noudattamisessa ja painonhallinnassa ravitsemusterapeutilta sekä lääkäriltä. Normaalipainoiset henkilöt eivät osallistu laihdutusinterventioon.

Tutkimuskäynnit:

1. Ensimmäisellä tutkimuskäynnillä tutkittavilta mitataan yön yli kestäneen paaston jälkeen paino, pituus, vyötärön- ja lantionympärysmitat, kehonkoostumus bioimpedanssilla, energiankulutus epäsuoralla kalorimetrillä sekä otetaan verinäyte vapaiden rasvahappojen, glyserolin, glukoosin, insuliinin, leptiinin, adiponektiinin, greliinin, TNF-α:n ja CRP:n määritystä varten. Lisäksi otetaan DNA-näyte tutkittavien sekvenssimuutosten määrittämiseksi. Tutkittavilta otetaan rasvakudosnäyte ja tehdään suonen sisäinen glukoosirasitustesti (frequently sampled intravenous glucose tolerance test, FSIGT). Tutkittavat aloittavat ENE-ruokavalion ravitsemusterapeutin ohjauksessa.

2. ENE-ruokavalion toteutuksen seurantakäynti viikon kuluttua ensimmäisestä käynnistä. Käynnillä mitataan yön yli kestäneen paaston jälkeen paino. Tutkittavilla on mahdollisuus tavata ravitsemusterapeutti.
3. ENE-ruokavalion toteutuksen seurantakäynti kolmen viikon kuluttua ensimmäisestä käynnistä. Käynnillä mitataan yön yli kestäneen paaston jälkeen paino. Tutkittavilla on mahdollisuus tavata ravitsemusterapeutti.
4. ENE-ruokavalio lopetetaan neljännellä tutkimuskäynnillä, kuuden viikon kuluttua ruokavalioon aloittamisesta. Tutkittavilta mitataan yön yli kestäneen paaston jälkeen paino, pituus, vyötärön- ja lantionympärysmitat, kehonkoostumus bioimpedanssilla, energiankulutus epäsuoralla kalorimetrillä sekä otetaan verinäyte terveydentilan arvioimiseksi (pieni verenkuva ja glukoosi). Epäsuoralla kalorimetrilla mitatun energiankulutuksen perusteella lasketaan yksilöllinen energiamäärä (1200 – 1600 kcal) seuraavan kolmen kuukauden laihdutusruokavaliota varten. Tutkittavat saavat ravitsemusterapeutin ohjauksen ruokavalion toteutukseen.
5. Laihdutuksen seurantakäynti kuukauden kuluttua yksilöllisen ruokavalion aloituksesta. Käynnillä mitataan yön yli kestäneen paaston jälkeen paino. Tutkittavilla on mahdollisuus tavata ravitsemusterapeutti.
6. Laihdutuksen seurantakäynti kahden kuukauden kuluttua yksilöllisen ruokavalion aloituksesta. Käynnillä mitataan yön yli kestäneen paaston jälkeen paino. Tutkittavilla on mahdollisuus tavata ravitsemusterapeutti.
7. Laihdutuksen seurantakäynti kolmen kuukauden kuluttua yksilöllisen ruokavalion aloituksesta, jolloin siirrytään painon ylläpitovaiheeseen. Tutkittavilta mitataan yön yli kestäneen paaston jälkeen paino, pituus, vyötärön- ja lantionympärysmitat, kehonkoostumus bioimpedanssilla, energiankulutus epäsuoralla kalorimetrillä sekä otetaan verinäyte vapaiden rasvahappojen, glyserolin, glukoosin ja insuliinin määritystä varten. Energiankulutuksen perusteella lasketaan yksilöllinen energiamäärä seuraavan kolmen kuukauden ylläpitojaksoa varten. Tutkittavat saavat ravitsemusterapeutin ohjauksen ruokavalion toteutukseen.
8. Ylläpitovaiheen seurantakäynti kuukauden kuluttua sen aloituksesta. Käynnillä mitataan yön yli kestäneen paaston jälkeen paino. Tutkittavilla on mahdollisuus tavata ravitsemusterapeutti.
9. Ylläpitovaiheen seurantakäynti kahden kuukauden kuluttua sen aloituksesta. Käynnillä mitataan yön yli kestäneen paaston jälkeen paino. Tutkittavilla on mahdollisuus tavata ravitsemusterapeutti.

10. Ylläpitovaiheen seurantakäynti kolmen kuukauden kuluttua aloituksesta, jolloin siirrytään tutkimuksen seurantavaiheeseen. Tutkittavilta mitataan yön yli kestäneen paaston jälkeen paino, pituus, vyötärön- ja lantionympärysmitat, kehonkoostumus bioimpedanssilla, energiankulutus epäsuoralla kalorimetrillä sekä otetaan verinäyte vapaiden rasvahappojen, glyserolin, glukoosin, insuliinin, adiponektiinin, greliinin, TNF-α:n ja CRP:n määritystä varten. Tutkittavilta otetaan rasvakudosnäyte ja tehdään FSIGT. Tutkittavilla on mahdollisuus tavata ravitsemusterapeuttia keskustellakseen saavuttamansa painon ylläpidosta seurantavaiheen aikana.
11. Seurantakäynti kolmen kuukauden kuluttua seurantavaiheen aloituksesta. Käynnillä mitataan yön yli kestäneen paaston jälkeen paino.
12. Seurantakäynti kuuden kuukauden kuluttua seurantavaiheen aloituksesta. Käynnillä mitataan yön yli kestäneen paaston jälkeen paino. Tutkittavilla on mahdollisuus tavata ravitsemusterapeutti.
13. Seurantakäynti yhdeksän kuukauden kuluttua seurantavaiheen aloituksesta. Käynnillä mitataan yön yli kestäneen paaston jälkeen paino.
14. Seurantakäynti vuoden kuluttua seurantavaiheen aloituksesta, jolloin tutkimus päättyy. Tutkittavilta mitataan yön yli kestäneen paaston jälkeen paino, pituus, vyötärön- ja lantionympärysmitat, kehonkoostumus bioimpedanssilla, energiankulutus epäsuoralla kalorimetrillä sekä otetaan verinäyte vapaiden rasvahappojen, glyserolin, glukoosin, insuliinin, adiponektiinin, greliinin, TNF-α:n ja CRP:n määritystä varten. Tutkittavilta otetaan rasvakudosnäyte ja FSIGT. Tutkittavilla on mahdollisuus tavata ravitsemusterapeutti keskustellakseen saavuttamansa painon ylläpidosta.

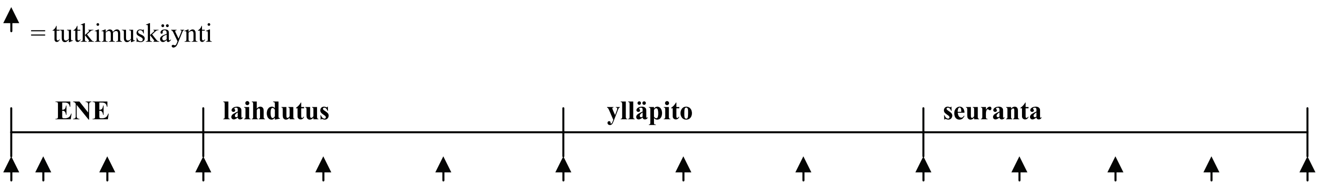

Normaalipainoiset vertailuhenkilöt käyvät kahdella tutkimuskäynnillä:

1. Ensimmäisellä tutkimuskäynnillä tutkittavilta mitataan yön yli kestäneen paaston jälkeen paino, pituus, vyötärön- ja lantionympärysmitat, kehonkoostumus bioimpedanssilla, energiankulutus epäsuoralla kalorimetrillä sekä otetaan verinäyte vapaiden rasvahappojen,

glyserolin, glukoosin, insuliinin, leptiinin, adiponektiinin, greliinin, TNF-α:n ja CRP:n määritystä varten. Lisäksi otetaan DNA-näyte tutkittavien sekvenssimuutosten määrittämiseksi. Tutkittavilta otetaan rasvakudosnäyte ja tehdään suonen sisäinen glukoosirasitustesti (frequently sampled intravenous glucose tolerance test, FSIGT).

2. Tutkittavat käyvät toisella tutkimuskäynnillä kuudesta kahdeksaan kuukauden kuluttua ensimmäisestä käynnistä (IGT-potilaiden seurantavaihen alku). Tällöin heille tehdään samat mittaukset ja toimenpiteet kuin ensimmäisellä kerrallakin.

MENETELMÄT

Verinäytteet

Verinäytteet analysoidaan KYS:n kliinisen kemian laboratoriossa, Kuopion yliopiston kliinisen ravitsemustieteen laitoksella ja kliinisen tutkimusyksikössä. FSIGT määritetään insuliiniherkkyyden selvittämiseksi. FSIGT:n aluksi kumpaankin kyynärtaipeen laskimoon asetaan suoneensisäiset kanyylit, joiden avulla otetaan paastonäytteet. Glukoosiliuos (300mg/kg) annostellaan kanyyliin 50 % liuoksena 1,5 minuutin kuluessa, minkä jälkeen kanyyli huuhdellaan 10 ml:lla 0,9 % suolaliuosta. 0,9 % suolaliuosta infusoidaan hiljalleen, kunnes 0,03 U/kg insuliinia annostellaan nopeasti kanyylin kautta laskimoon 20 minuutin kuluttua glukoosiannoksesta. Suolaliuoksen infuusiota jatketaan 1,5 minuuttia insuliinin annostelun jälkeen. Plasman glukoosi- ja insuliinipitoisuuksien määrittämiseksi otetaan verinäytteet ennen glukoosin annostelua (–5 ja 0 minuuttia) sekä 23 kertaa annoksen jälkeen (2, 4, 6, 8, 10, 12, 14, 16, 19, 22, 24, 27, 30, 40, 50, 60, 70, 90, 100, 120, 140, 160 ja 180 minuuttia) kanyylin kautta. Glukoosi- ja insuliininäytteet analysoidaan kliinisen ravitsemustieteen laitoksella. Tulokset analysoidaan laskemalla glukoosin oton tehokkuus (S_G) ja insuliiniherkkyysindeksi (S_I) käyttäen Minmod ohjelmaa. Lisäksi analysoidaan 1.vaiheen insuliinivaste (AIR) laskemalla perustason ylimenevän insuliinitason pinta-ala 0 - 10 minuuttia.

Antropometria ja perusenergiankulutus

Paino mitataan puolen kilon tarkkuudella sekä pituus, vyötärön- ja lantionympärysmitat yhden senttimetrin tarkkuudella. Rasvaprosentti määritetään bioelektrisellä impedanssilla. Perusenergiankulutus mitataan epäsuoralla kalorimetrilla.

DNA-näytteiden analyysit

DNA-näytteet säilytetään –70ºC:ssa, kunnes ne analysoidaan. DNA eristetään verinäytteen valkosoluista suolasaostusmenetelmällä (Miller *et al.*, 1988). Tutkittavat geenialueet monistetaan polymeraasiketjureaktiolla (PCR). Uusien sekvenssimuutosten etsimiseen käytetään single-strand conformation polymorphism (SSCP) menetelmää. Tunnettuja, jo aiemmin raportoituja geenivariantteja, tutkitaan restriktioentsyymien käyttöön perustuvan RFLP (restriction fragment length polymorphism)-menetelmän avulla. RFLP-menetelmän rinnalla käytetään myös ns. SNP (single nucleotid polymorphism)-detektiomenetelmää. Tutkittavat geenit liittyvät kehon energia-, glukoosi- ja insuliiniaineenvaihduntaan sekä rasvakudoksen aineenvaihduntaan, rasvan varastoitumiseen sekä vapautumiseen rasvakudoksesta. Tutkittavia geenejä ovat mm. PPAR-γ, adrenergiset reseptorit (α2b, β2, β3), leptiini, leptiinireseptori, SREBP, adiponektiini, melanokortiini-4 reseptori, greliini, resistiini, aldoosireduktaasi, irtikytkijäproteiinit (UCPt), hormonisensitiivinen lipaasi, lipoproteiinilipaasi, tuumorinekroositekijä α, interleukiini 6 ja fosfoinositoli 3-kinaasi. Lisäksi tutkitaan aiemmin tuntemattomia geenejä käyttäen hyväksi geenikirjastojen sisältämiä ihmisen geenialueita.

Rasvakudosnäyte ja geenien ilmentymisen tutkiminen

Rasvakudosnäytteet otetaan mahalta suoliluunharjanteen ja navan puolivälistä ja lantiolta reisiluun pään kohdalta neula- tai avonäytteinä. Näytteet käsitellään RNA-Laterilla©, joka stabiloi RNA:n. Tämän jälkeen kudosnäytteitä säilytetään –70ºC:ssä näytteiden analysointiin asti, jotta kudoksen RNA säilyisi mahdollisimman muuttumattomana. Näytteet otetaan paikallispuudutuksessa.

Rasvakudosnäytteestä eristetään kokonais-RNA kaupallisella kitillä, jonka jälkeen RNA käännetään komplementaariseksi DNA:ksi. Geenien ilmentymistä rasvakudoksessa tutkitaan DNA-mikrosirumenetelmän (Moreno-Aliaga *et al.*, 2001), kvantitatiivisen polymeraasiketjureaktiomenetelmän (Bustin, 2002) sekä kompetitiivisen käänteisen polymeraasiketjureaktiomenetelmän (Auboeuf ja Vidal 1997) avulla .

Geenit, joiden ilmentymistä tutkitaan, liittyvät mm. insuliini-, glukoosi- ja energia-aineenvaihduntaa sekä rasvakudoksen aineenvaihduntaan, rasvan varastoitumiseen sekä vapautumiseen rasvakudoksesta. Tutkittavia geenejä ovat mm. PPAR-γ, adrenergiset reseptorit (α2b, β2, β3), leptiini, leptiinireseptori, SREBP, adiponektiini, melanokortiini-4 reseptori, greliini, resistiini, aldoosireduktaasi, irtikytkijäproteiinit (UCPt), hormonisensitiivinen lipaasi, lipoproteiinilipaasi, tuumorinekroositekijä α, interleukiini 6 ja fosfoinositoli 3-kinaasi. Lisäksi

tutkitaan aiemmin tuntemattomien geenien ilmentymisen muutoksia käyttäen hyväksi geenikirjastojen sisältämiä ihmisen geenialueita.

NÄYTTEIDEN ANONYMISOINTI

Näytteet koodataan siten, että vain vastuullinen tutkija voi yhdistää tutkimuksessa saatavat tiedot (DNA-, RNA- ja verinäyteanalyysit) toisiinsa. Näytteet tullaan koodaamaan säilytystä varten numeroin, joita käytetään kaikissa saman potilaan näytteissä. Tutkittavakohtaisia tietoja ei tulla missään tapauksessa luovuttamaan sivullisille.

POTILAILLE ANNETTAVAT TIEDOT

Tutkittavat saavat tietoonsa heiltä otettujen verinäytteiden, perusenergiankulutus- ja kehonkoostumusmittausten tulokset. Tutkimuksessa määritettävät geenit ja niiden ilmentyminen eivät sinällään aiheuta lihavuutta, vaan ne saattavat olla lihavuudelle altistavia tekijöitä. Tällaisessa tutkimuksessa yksittäisiin perintötekijöihin liittyvien poikkeavuuksien kliinistä merkitystä ei aina voida arvioida. DNA- tai RNA-näytteitä ei myöskään käytetä sairauksien diagnosointiin. Tämän vuoksi DNA- ja RNA-analyyseihin liittyviä tuloksia ei tulla kertomaan tutkittaville, sillä tulokset ovat yksilökohtaisesti vaikeasti tulkittavia. Nykytietämyksen valossa on myös epätodennäköistä, että näillä tiedoilla olisi merkitystä tutkittavan terveydelle, varsinkin kun lihavuudelle altistavat tekijät ovat moninaiset niin perintötekijöiden kuin ympäristötekijöidenkin osalta. Maailmassa on vain muutamia tapauksia, joissa yksittäisen geenin muutos on aiheuttanut lihavuuden ihmiselle. Sen sijaan ihmisen tavanomaiselle lihavuudelle altistavia geenejä tiedetään olevan lukuisia, eikä yksikään niistä aiheuta lihavuutta ilman ympäristötekijöiden myötävaikutusta. Mikäli joku tutkittavista välttämättä haluaa tietoonsa häntä koskevien perintötekijämääritysten tulokset, hänen kanssaan keskustellaan tuloksista ja selostetaan tämän hetkisen kirjallisuuden valossa tulosten merkitys hänen terveytensä kannalta.

AIKATAULU

Aineiston seulonta ja keruu aloitetaan keväällä 2003. Arviomme on, että aineiston keruu kestää noin vuoden. Näin ollen analyysit voidaan aloittaa vuoden 2004 aikana ja tulokset raportoidaan 2005-2006 aikana kansainvälisissä lääke- ja ravitsemustieteellisissä lehdissä.

TUTKIMUKSEN MERKITYS

Lihavuus ja tyypin 2 diabetes ovat yleistyviä terveysongelmia sekä kehittyneissä että kehittyvissä maissa (WHO, 2000). Epidemian pysäyttämiseksi tarvitsemme nopeasti uutta tietoa sekä

elämäntapatekijöiden että perimän merkityksestä lihavuuden synnyssä. Laihdutus korjaa selvästi tyypin 2 diabeteksen keskeistä poikkeavuutta, insuliiniresistenssiä, joten rasvakudoksen merkitys myös insuliiniresistenssin ja tyypin 2 diabeteksen synnyn kannalta on keskeinen. Tutkimuksen lähestymistapa antaa uutta tietoa lihavuuden ja insuliiniresistenssin kannalta keskeisten geenien toiminnasta lisäten lihavuuden syiden entistä syvällisempää tuntemusta ja mahdollistaen nykyistä tehokkaammin niiden henkilöiden tunnistamisen, joilla on vahva geneettinen taipumus lihavuuteen tai sen aiheuttamiin komplikaatioihin. Tällöin myös lihavuuden ehkäisyä ja lihavuuden uusien hoitomuotojen kehittämistä voidaan tehostaa.

VIITTEET

Auboeuf D, Vidal H (1997) The use of the reverse transcriptase-competitive polymerase chain reaction to investigate the in vivo regulation of gene expression in small tissue samples. *Ann Biochem* **245**, 141-148.

Bustin SA (2002) Quantification of mRNA using real-time reverse transcription PCR (RT-PCR): trends and problems. *J Mol Endocrinol* **29**, 23-39.

Ebbeling CB, Pawlak DB & Ludwig DS (2002) Childhood obesity: public-health crisis, common sense cure. *Lancet* **360**, 473-482.

Heinonen P, Koulu M, Pesonen U, Karvonen MK, Rissanen A, Laakso M, Valve R, Uusitupa M & Scheinin M (1999) Identification of a three-amino acid deletion in the alpha2B-adrenergic receptor that is associated with reduced basal metabolic rate in obese subjects. *J Clin Endocrinol Metab* **84**, 2429-2433.

Kallio J, Pesonen U, Kaipio K, Karvonen MK, Jaakkola U, Heinonen OJ, Uusitupa MI & Koulu M (2001) Altered intracellular processing and release of neuropeptide Y due to leucine 7 to proline 7 polymorphism in the signal peptide of preproneuropeptide Y in humans. *Faseb J* **15**, 1242-1244.

Karhunen L, Franssila-Kallunki A, Rissanen P, Valve R, Kolehmainen M, Rissanen A & Uusitupa M (2000) Effect of orlistat treatment on body composition and resting energy expenditure during a two-year weight-reduction programme in obese Finns. *Int J Obes Relat Metab Disord* **24**, 1567-1572.

Karvonen MK, Pesonen U, Koulu M, Niskanen L, Laakso M, Rissanen A, Dekker JM, Hart LM, Valve R & Uusitupa MI (1998) Association of a leucine(7)-to-proline(7) polymorphism in the signal peptide of neuropeptide Y with high serum cholesterol and LDL cholesterol levels. *Nat Med* **4**, 1434-1437.

Kim S & Moustaid-Moussa N (2000) Secretory, endocrine and autocrine/paracrine function of the adipocyte. *J Nutr* **130**, 3110S-3115S.

Kolehmainen M, Ohisalo JJ, Kaartinen JM, Tuononen V, Paakkonen M, Poikolainen E, Alhava E & Uusitupa MI (2000) Concordance of in vivo microdialysis and in vitro techniques in the studies of adipose tissue metabolism. *Int J Obes Relat Metab Disord* **24**, 1426-1432.

Kolehmainen M, Vidal H, Alhava E & Uusitupa MI (2001) Sterol regulatory element binding protein 1c (SREBP-1c) expression in human obesity. *Obes Res* **9**, 706-712.

Kolehmainen M, Vidal H, Ohisalo JJ, Pirinen E, Alhava E & Uusitupa MI (2002) Hormone sensitive lipase expression and adipose tissue metabolism show gender difference in obese subjects after weight loss. *Int J Obes Relat Metab Disord* **26**, 6-16.

Kopelman PG (2000) Obesity as a medical problem. *Nature* **404**, 635-643.

Lahti-Koski M, Vartiainen E, Mannisto S & Pietinen P (2000) Age, education and occupation as determinants of trends in body mass index in Finland from 1982 to 1997. *Int J Obes Relat Metab Disord* **24**, 1669-1676.

Lindi V, Sivenius K, Niskanen L, Laakso M & Uusitupa MI (2001) Effect of the Pro12Ala polymorphism of the PPAR-gamma2 gene on long-term weight change in Finnish non-diabetic subjects. *Diabetologia* **44**, 925-926.

Lindi VI, Uusitupa MI, Lindstrom J, Louheranta A, Eriksson JG, Valle TT, Hamalainen H, Ilanne-Parikka P, Keinanen-Kiukaanniemi S, Laakso M & Tuomilehto J (2002) Association of the Pro12Ala polymorphism in the PPAR-gamma2 gene with 3-year incidence of type 2 diabetes and body weight change in the Finnish Diabetes Prevention Study. *Diabetes* **51**, 2581-2586.

Miller SA, Dykes DD & Polesky HF (1988) A simple salting out procedure for extracting DNA from human nucleated cells. *Nucleic Acids Research* **16**, 1215.

Moreno-Aliaga MJ, Marti A, Garcia-Foncillas J & Alfredo Martinez J (2001) DNA hybridization arrays: a powerful technology for nutritional and obesity research. *Br J Nutr* **86**, 119-122.

Puska P, Helakorpi S, Prättälä R & Uutela A (1996) Suomalaisten painokäyrä noususuunnassa - aikuisväestön terveyskäyttäytymisseurannan tuloksia. *Suom Lääkäril* **30**, 3123-3128.

Rocchi S & Auwerx J (1999) Peroxisome proliferator-activated receptor-gamma: a versatile metabolic regulator. *Ann Med* **31**, 342-351.

Sipiläinen R, Uusitupa M, Heikkinen S, Rissanen A & Laakso M (1997a) Polymorphism of the beta3-adrenergic receptor gene affects basal metabolic rate in obese Finns. *Diabetes* **46**, 77-80.

Sipiläinen R, Uusitupa M, Heikkinen S, Rissanen A & Laakso M (1997b) Variants in the human intestinal fatty acid binding protein 2 gene in obese subjects. *J Clin Endocrinol Metab* **82**, 2629-2632.

Sivenius K, Lindi V, Niskanen L, Laakso M & Uusitupa M (2001) Effect of a three-amino acid deletion in the alpha2B-adrenergic receptor gene on long-term body weight change in Finnish non-diabetic and type 2 diabetic subjects. *Int J Obes Relat Metab Disord* **25**, 1609-1614.

Sivenius K, Valve R, Lindi V, Niskanen L, Laakso M & Uusitupa M (2000) Synergistic effect of polymorphisms in uncoupling protein 1 and beta3-adrenergic receptor genes on long-term body weight change in Finnish type 2 diabetic and non-diabetic control subjects. *Int J Obes Relat Metab Disord* **24**, 514-519.

Tuomilehto J, Lindström J, Eriksson JG, Valle TT, Hämäläinen H, Ilanne-Parikka P, Keinänen-Kiukaanniemi S, Laakso M, Louheranta A, Rastas M, Salminen V & Uusitupa M (2001) Prevention of type 2 diabetes mellitus by changes in lifestyle among subjects with impaired glucose tolerance. *N Engl J Med* **344**, 1343-1350.

Valve R, Heikkinen S, Rissanen A, Laakso M & Uusitupa M (1998) Synergistic effect of polymorphisms in uncoupling protein 1 and beta3-adrenergic receptor genes on basal metabolic rate in obese Finns. *Diabetologia* **41**, 357-361.

Valve R, Sivenius K, Miettinen R, Pihlajamaki J, Rissanen A, Deeb SS, Auwerx J, Uusitupa M & Laakso M (1999) Two polymorphisms in the peroxisome proliferator-activated receptor-gamma gene are associated with severe overweight among obese women. *J Clin Endocrinol Metab* **84**, 3708-3712.

WHO (2000) Obesity: preventing and managing the global epidemic. Report of a WHO consultation. *World Health Organ Tech Rep Ser* **894**, i-xii, 1-253.

Liite 1

POTILASTIEDOTE JA –SUOSTUMUS

Lihavuudelle ja insuliiniresistenssille mahdollisesti altistavien perintötekijöiden ilmentyminen rasvakudoksessa

Kuopion yliopiston kliinisen ravitsemustieteen laitoksella ja Elintarvikkeiden terveysvaikutusten tutkimuskeskuksessa selvitetään lihavuudelle ja insuliinin tehottomuudelle elimistössä mahdollisesti altistavissa perintötekijöissä havaittujen muutosten vaikutusta näiden perintötekijöiden toimintaan rasvakudoksessa. Pyydämme Teitä perehtymään tähän tiedotteeseen ja luettuanne sen antamaan suostumuksenne tutkimukseen osallistumisesta. Jos Teillä on kysyttävää tutkimuksesta, vastaan mielelläni kysymyksiinne: **Marjukka Kolehmainen THM/ Kuopion yliopisto, kliinisen ravitsemustieteen laitos puh. 017-162735 tai Titta Salopuro FM/ Kuopion yliopisto, kliinisen ravitsemustieteen laitos puh. 017-162769 tai sähköpostitse: marjukka.kolehmainen@uku.fi tai titta.salopuro@uku.fi.**

Lihavuuden taustalla vaikuttavat monet tekijät, ja lihavuus onkin useiden ympäristötekijöiden ja perintötekijöiden sekä niiden yhdysvaikutusten seurausta. Tiedetään, että ravinto vaikuttaa perintötekijöiden toimintaan solutasolla, ja toisaalta perintötekijät vaikuttavat ravintoaineiden imeytymiseen, niiden aineenvaihduntaan elimistössä ja energiantuottoon. Toistaiseksi perintötekijöiden ja ruokavalion yhdysvaikutuksista ei ole paljoakaan tutkimustietoa. Uusien tutkimusmenetelmien kehittäminen mahdollistaa tänä päivänä yhdysvaikutusten tehokkaamman tutkimisen.

Rasvakudos on osoittautunut viimeaikaisten tutkimusten perusteella aktiiviseksi kudokseksi, joka osallistuu energia-, glukoosi- ja insuliiniaineenvaihdunnan säätelyyn tuottamalla eräitä merkittäviä tekijöitä, jotka vaikuttavat perintötekijöiden ilmentymiseen rasvakudoksessa. Lisäksi rasvakudoksesta vapautuu verenkiertoon tekijöitä, joiden epäillään olevan yhteydessä sekä lihavuuden että insuliiniresistenssin syntyyn. Tämän vuoksi rasvakudoksen tutkiminen on tärkeä osa ei ainoastaan lihavuuden vaan myös aikuistyypin sokeritaudin taustalla vaikuttavien tekijöiden selvitystyötä.

Tässä tutkimuksessa selvitettävät perintötekijät vaikuttavat kehon energia- ja sokeriaineenvaihduntaan sekä rasvakudoksen aineenvaihduntaan. Näissä perintötekijöissä on löydetty muutoksia, joiden on ajateltu liittyvän perinnölliseen alttiuteen lihoa. Ne EIVÄT kuitenkaan aiheuta lihomista sinällään !

Tarjoamme teille mahdollisuutta osallistua tähän tutkimukseen. Tutkimukseen osallistuvat kutsutaan Elintarvikkeiden terveysvaikutusten tutkimuskeskukseen Kuopion yliopiston kliinisen ravitsemustieteen laitokselle seulontaan, jonka aikana heiltä mitataan yön yli kestäneen paaston jälkeen paino ja pituus sekä otetaan verinäyte pienen verenkuvan, LDL-kolesterolin, HDL-kolesterolin, triglyseridien, glukoosin, insuliinin ja TSH:n määrittämistä varten. Lisäksi tehdään sokerirasitustesti, jossa nautitaan 75 g glukoosia sekoitettuna 3 dl:aan vettä ja otetaan glukoosi- ja insuliininäytteet 0, 60 ja 120 min kuluttua glukoosiliuoksen nauttimisesta. Varsinaiseen tutkimukseen osallistuville ilmoitetaan erikseen.

Tutkittavat noudattavat aluksi 6 viikkoa erittäin niukasti energiaa sisältävää (400 – 600 kcal) ruokavaliota (=ENE) täysipainoisen valmisteena avulla. ENE-jakson loputtua, alkaa yksilöllinen 1200 – 1600 kcal:a sisältävä laihdutusruokavalio, jota noudatetaan 3 kuukauden ajan. Laihdutuksen

jälkeen tutkittavat siirtyvät 3 kuukautta kestävä ylläpitovaiheelle, jonka tarkoituksena on pitää saavutettu paino ennallaan. Tämän jakson loputtua tutkittavia seurataan vuoden ajan (=seurantajakso), jolloin tutkittavat käyvät painonmittauksessa 3 kuukauden välein. Tutkittavat saavat ravitsemusterapeutin ohjausta ruokavalion ja painonhallinnan toteuttamiseksi. Tutkimuskäyntejä on yhteensä 14. Jokaisen tutkimusjakson (ENE, yksilöllinen laihdutus, ylläpito ja seuranta) aloittavalla sekä koko tutkimuksen lopettavalla käynnillä (yht. 5 kpl) tutkittavilta mitataan yön yli kestäneen paaston jälkeen paino, pituus, vyötärön- ja lantionympärysmitat, kehonkoostumus bioimpedanssilla, energiankulutus epäsuoralla kalorimetrillä sekä otetaan verinäyte. Ensimmäisellä tutkimuskäynnillä otetaan myös verinäyte DNA-analyyseja varten.

Tutkimuksen aluksi, seurantajaksolle siirryttäessä ja tutkimuksen lopuksi (3 kertaa) tutkittavilta otetaan rasvakudosnäyte avo- tai neulanäytteenä mahan- ja lantionalueen ihonalaisesta rasvakudoksesta. Mikäli näytteet otetaan avonäytteinä, näytteet ottokohtaan laitetaan 3 – 5 tikkiä, jotka tulee poistattaa myöhemmin sovittuna ajankohtana. Lisäksi näillä käynneillä tehdään suonen sisäinen glukoosirasitustesti. Rasitustestin aluksi kumpaankin kyynärtaipeen laskimoon asetetaan suoneensisäiset kanyylit, joiden avulla otetaan paastonäytteet. Toiseen kanyyliin annostellaan sokeriliuos (300mg/kg) 50 % liuoksena 1,5 minuutin kuluessa, minkä jälkeen kanyyli huuhdellaan. 20 minuutin kuluttua sokeriliuoksesta 0,03 U/kg insuliinia annostellaan nopeasti kanyylin kautta laskimoon. Glukoosi- ja insuliinipitoisuuksien määrittämiseksi otetaan verinäytteet ennen glukoosin annostelua (–5 ja 0 minuuttia) sekä 23 kertaa annoksen jälkeen (2, 4, 6, 8, 10, 12, 14, 16, 19, 22, 24, 27, 30, 40, 50, 60, 70, 90, 100, 120, 140, 160 ja 180 minuuttia) kanyylin kautta, erillisiä pistämisiä ei tarvita.

Muut käynnit (9 kpl) ovat kontrollikäyntejä, jolloin tutkittavilta mitataan paino ja heillä on mahdollisuus tavata ravitsemusterapeuttia.

Normaalipainoiset vertailuhenkilöt käyvät kahdella tutkimuskäynnillä n. 6 – 8 kuukauden välein, jolloin heille tehdään samat tutkimukset kuin ylipainoisille tutkittaville tutkimuksen aloituskäynnillä. Heiltä otetaan myös näillä käynneillä rasvakudosnäyte ja tehdään suonen sisäinen glukoosirasitustesti.

Rasvakudosnäytteistä tutkitaan perintötekijöiden ilmentymistä. Nämä sekä DNA-analyyseillä selvitettävät perintötekijät liittyvät kehon energia-, glukoosi- ja insuliiniaineen-vaihduntaan sekä rasvakudoksen aineenvaihduntaan, rasvan varastoitumiseen sekä vapautumiseen rasvakudoksesta. Rasvakudos- ja verinäytteet koodataan säilytystä varten niin, ettei niistä määritettäviä tietoja voida yhdistää henkilötietoihinne suoraan. Ainoastaan vastuullinen tutkija saa tietoonsa koodin, jonka avulla hän voi yhdistää tutkimuksesta saatavat tiedot toisiinsa. Tuloksia ei missään vaiheessa raportoida yksilökohtaisesti, vain ryhmätasolla. Näytteet säilytetään kliinisen ravitsemustieteen laitoksella tutkimuksen keston ajan, eli 3 - 5 vuotta, jonka jälkeen ne hävitetään muiden ns. riskijätteiden mukana yliopistolta. Tutkittavat saavat käyttöönsä kehonkoostumusta sekä veriarvoja selvittävien mittausten tulokset. Sen sijaan kudos- ja verinäytteistä analysoitavien perintötekijöiden tiedot jäävät vain tutkimusryhmän käyttöön. Näin menetellään sen vuoksi, että näihin perintötekijöihin liittyvät tulokset ovat vaikeasti tulkittavia yksilöllisesti, ja on hyvin epätodennäköistä, että tuloksilla olisi merkitystä tutkittavien terveydelle.

Teille on selvitetty tutkimukseen liittyviä yksityiskohtia. Kun olette saanut mielestänne riittävästi tietoa ja vastaukset mahdollisiin kysymyksiinne, pyydän Teitä allekirjoittamaan suostumuslomakkeen. Tutkimukseen osallistuminen on vapaaehtoista ja voitte keskeyttää tutkimuksen, milloin haluatte syytä ilmoittamatta. Tutkimustietonne ovat ehdottoman luottamuksellisia, eikä niitä anneta sivullisille. Tutkimuksen aikana voitte milloin tahansa ottaa yhteyttä tutkijaan, jos tarvitsette tietoa tai neuvoa tutkimukseen liittyen.

Liite 2

POTILASSUOSTUMUS

Osallistun vapaaehtoisesti lihavuuteen ja insuliiniresistenssiin mahdollisesti liittyvien perintötekijöiden ilmentymistä rasvakudoksessa selvittävään tutkimukseen. Olen saanut sekä suullista että kirjallista tietoa tutkimuksen tavoitteista ja menetelmistä. Tiedän, että voin lopettaa tutkimukseen syytä ilmoittamatta ilman, että se vaikuttaa jatkossa hoitosuhteeseeni.

Tutkittavan nimi:

Syntymäaika:

Tutkittavan allekirjoitus, aika ja paikka:

Nimenselvennys:

Tutkijan allekirjoitus, aika ja paikka:

Nimenselvennys:

Liite 3

KUSTANNUSARVIO:

Kustannusarvion yhteissumma on laskettu 120 tutkimukseen osallistuvalle henkilölle:

Verinäyte:	CRP	1200
	glukoosi	1220
	insuliini	5416
	leptiini	4165
	vapaat rasvahapot	4664
	glyseroli	3960
	pieni verenkuva	1230
	kokonaiskolesteroli, HDL-kolesteroli, LDL-kolesteroli, triglyseridit yht.	1070
	greliini	4080
	adiponektiini	5100
	TNF-α	5100
	γ-GT	400
Perusenergiankulutus		9126
Kehonkoostumus bioimpedanssilla		4590
Rasvakudosnäytteenotto		51000
RNA:n eristys 6000 € (kitit, reagenssit)		6000
DNA:n eristys 5000 € (kitit, reagenssit)		5000
DNA-mikrosirumenetelmä 20100 € (lasit, reagenssit)		20100
Kvantitatiivinen PCR 3000 € (reagenssit)		3000
RT-kompetetiivinen-PCR		5100

Yhteensä **141 521 €**
